# Depletion of Specific Cell Populations by Complement Depletion

**DOI:** 10.3791/1487

**Published:** 2010-02-05

**Authors:** Bonnie N. Dittel

**Affiliations:** BloodCenter of Wisconsin, Blood Research Institute

## Abstract

The purification of immune cell populations is often required in order to study their unique functions.  In particular, molecular approaches such as real-time PCR and microarray analysis require the isolation of cell populations with high purity.  Commonly used purification strategies include fluorescent activated cell sorting (FACS), magnetic bead separation and complement depletion.  Of the three strategies, complement depletion offers the advantages of being fast, inexpensive, gentle on the cells and a high cell yield.  The complement system is composed of a large number of plasma proteins that when activated initiate a proteolytic cascade culminating in the formation of a membrane-attack complex that forms a pore on a cell surface resulting in cell death^1^.  The classical pathway is activated by IgM and IgG antibodies and was first described as a mechanism for killing bacteria.  With the generation of monoclonal antibodies (mAb), the complement cascade can be used to lyse any cell population in an antigen-specific manner.  Depletion of cells by the complement cascade is achieved by the addition of complement fixing antigen-specific antibodies and rabbit complement to the starting cell population.  The cells are incubated for one hour at 37°C and the lysed cells are subsequently removed by two rounds of washing.  MAb with a high efficiency for complement fixation typically deplete 95-100% of the targeted cell population.  Depending on the purification strategy for the targeted cell population, complement depletion can be used for cell purification or for the enrichment of cell populations that then can be further purified by a subsequent method.

**Figure:**
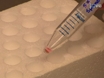


## Protocol

### A. Rabbit Complement

Stock rabbit complement should be thawed at room temperature, and then aliquoted into 0.5 and/or 1 ml volumes and frozen at -20-80  °C for long-term storage.
Each lot of complement should be titrated to determine the optimal working concentration. Typically, a dilution of 1:10-1:20 is optimal.
Rabbit complement is not always presterilized prior to sale, depending on the final use of the cells, the complement should be filtered sterilized when aliquoted and frozen or prior to addition to the cells.

### B. Antibody Selection

Not all antibodies are effective at complement activation, thus the antibodies must first be tested for activity.
Both mouse and rat mAb are effective at complement activation.
IgM antibodies are generally the most efficient for use in complement-induced cell lysis followed by IgG. The IgG isotypes best for complement activation vary between species.
Each antibody should be titrated for optimal use at a cell density of 10^7^ cells/ml.

### C. Basic Protocol

Adjust the starting cell population to 5 x 10^7^ cells/ml in medium containing heat inactivated FCS. The final volume of the cells will be twice the starting volume. Remember to save some cells so that the depletion can be quantified.
Each antibody should be added to the cell suspension at the proper predetermined concentration or dilution. Mix the cells well by inversion.
Calculate the amount of complement needed. Thaw the complement stock at room temperature. If the complement is not sterile, sterilize it using a low protein binding syringe tip filter. To the syringe, add several ml of medium prior to the addition of the complement and filter directly into the tube containing the cells and antibody.
Adjust the final volume in the tube so that the cells are at 1 x 10^7^ cells/ml.
Incubate the cells at 37  °C for one hour, mixing every 15 minutes.
Centrifuge the cells and discard the supernatant.
Wash with medium twice. Cell debris will stick to the side of polystyrene tubes. Dead cells and debris can be eliminated using a cell strainer or by density gradient centrifugation.
Check the cell purity by comparing the cell population of interest before and after depletion. Flow cytometry and immunohistochemistry are two good methods of determining cell purity.

### Representative Results

When using a mAb that is highly efficient at activating complement, cell depletion after one round is greater then 95%. This is shown in Figure 1 where we started with mouse splenocytes composed of 32.2% αβ T cells as detected by expression of TCRβ (eBioscience, San Diego, CA) by flow cytometry. We depleted T cells using a mAb specific for Thy1, a protein expressed by all T cells, but not other leukocytes. The antibody we used was Y-19 a rat IgG2c^2^. Following the procedure described, the TCRβ population was reduced to 1.56%, a greater than 95% reduction.


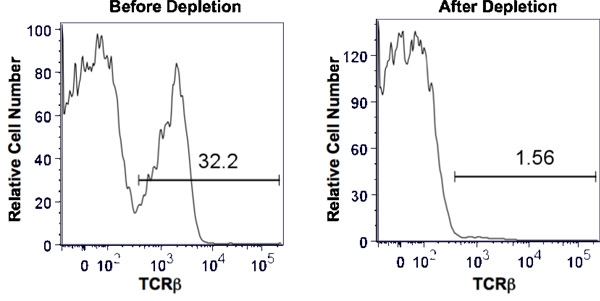
 **Figure 1. Depletion of thy1^+^ splenic T cells by complement activation.** Total mouse splenocytes were obtained by homogenization of the spleen and lysis of RBC. Anti-thy1 and baby rabbit complement at predetermined concentrations were added to a single cell suspension of splenocytes such that the final cell concentration was 1 x 10^7^ cells/ml. After incubation for 1 hour at 37 °C, the cells were washed twice and evaluated by trypan blue exclusion for cell viability. The efficiency of depletion was assessed by flow cytometry comparing the percentage of TCRβ+ cells before depletion (left panel) and after depletion (right panel). The horizontal bar indicates positive staining and the number above the bar is the percent positive.

## Discussion

The purification of cell populations is a common procedure required for the study of their functions. Here we describe a fast and effective method of depleting cell populations using antigen-specific antibodies and complement depletion. The depletion of any cell population can be achieved if a complement-activating antibody is available to a lineage specific marker. Here we demonstrated the depletion of T cells using a mAb specific for Thy1, a protein expressed by essentially all T cell populations. We depleted over 95% of αβ T cells and the remaining splenocytes were increased from 68% to 98%. This level of cell purity is similar to other purification strategies including FACS and magnetic bead separation. Complement depletion is a fast technique that can be performed in 1.5 hours once the starting cell population is obtained. Another advantage of complement depletion is that any laboratory can perform the technique because it does not require expensive equipment and reagents. The only major pieces of equipment required are a water bath and a centrifuge. Rabbit complement can be purchased from a variety of vendors at a reasonable cost (see table of reagents) and many mAb can be purchased from ATTC and grown locally to control costs. In contrast, FACS requires a flow cytometer with cell sorting capacity that will cost in the six figures and expensive fluorescently tagged mAb. Magnetic bead separation is moderately priced, but requires the purchase of a magnet and magnetic beads, which can be costly. In addition, some magnetic bead separation strategies also require the purchase of columns.

The main stumbling block to the use of complement for cell depletion is the availability of complement activating mAb with the antigen specificity required. Although human, mouse and rat antibodies all have the capacity to fix complement, some isotypes are better than others. Although IgM from all three species is highly effective at fixing complement the IgG isotypes vary. Thus every mAb must first be tested in a pilot study to determine its effectiveness. For antibodies that are not highly efficient, two rounds of complement depletion can be performed without affecting the viability of the remaining cell populations. As a cost savings, the antibodies should be titrated for optimal effectiveness. In addition, the antibodies used do not need to be purified. Hybridoma supernatants and ascites fluid work just as effectively, which is particularly convenient for IgM antibodies, which are difficult to purify. As with the antibodies, it is imperative that the complement be titrated in a pilot study. A too high of concentration will result in loss of viability of non-targeted cell populations. Also important is that the incubation time does not exceed 60 minutes.

In our protocol, we co-incubate the antibody and the complement together. However, the cells can be labeled with antibody for 15-30 minutes and washed prior to the addition of complement. When using this strategy, make sure to perform the antibody incubations on ice to prevent the loss of the cell antigen on the cell surface, as occurs with some proteins by patching and capping or receptor internalization. Although highly efficient, the main drawback to complement depletion as compared to other methods is that it cannot be used for positive selection. Thus it is difficult to obtain subpopulations of cells. For example, it would be difficult to purify marginal zone B cells from follicular B cells, which would best be done by FACS. In this situation, complement activation could be used to deplete T cells to reduce the number of cells being sorted. This strategy would reduce the sort time, which would save money and would be less harsh on the cells.

Since the purification of specific cell populations is a common requirement a number of strategies have been developed. The main advantages of the complement depletion strategy are its affordability, technical simplicity and time savings.
